# A CROSS SECTION OF SKIN DISEASES IN RURAL ALLAHABAD

**DOI:** 10.4103/0019-5154.44789

**Published:** 2008

**Authors:** Sanjiv Grover, Rakesh K Ranyal, Mehar K Bedi

**Affiliations:** *From the Department of Dermatology, Armed Forces Medical College, Pune - 411040, Maharashtra, India*; 1 *From the Director Medical Services (P) – 1A, O/o DGMS (Air), Delhi - 110 066, India*; 2 *From the SMO, AF Station, Palam, New Delhi, India*

**Keywords:** *Skin diseases pattern*, *rural*, *Allahabad*

## Abstract

**Background:**

The pattern of skin diseases varies form one country to another and across different parts within the same country. A two-day multispecialty medical camp was held among the local population at the district of Kausambi, Allahabad, UP, in October 2005. A cross section of pattern of skin diseases observed at the camp is reported and compared with similar studies in literature.

**Materials and Methods:**

All cases attending the medical camp were included in the study. All those with dermatological complaints were examined in detail, brief relevant history was elicited and clinical diagnosis was made.

**Results:**

Skin diseases comprised 7.86% of all those who attended the camp. The 11–20 year age group was the most common age group involved with 164 (31.4%) cases. Infective disorders were found in 59.1% and noninfective disorders in 40.9% of all the skin cases. Among the infective disorders, fungal infections were most common (54.52%), and among the noninfective dermatoses, eczemas were most common (39.2%) cutaneous disorders.

**Conclusion:**

Our study brought out a higher prevalence of infective dermatoses and a relatively higher, but statistically insignificant, prevalence of fungal infections, scabies and eczemas, thereby reflecting minor regional variance in our study group.

## Introduction

The pattern of skin diseases varies form one country to another and across different parts within the same country.^1^ In particular, in India, where customs, religions, languages, climate and socio-economic conditions vary across different parts of the country. Due to lack of education, patients may not report for treatment unless compelled by the severity of the symptoms. Up to 80% of the populace suffering from skin problems may not seek medical help.^1^ Knowledge of this hidden section of population is important as it can affect the delivery of health care.

A number of workers have reported different patterns of skin diseases in different geographical areas.^2^–^12^ We report a cross section of pattern of skin diseases observed at a medical camp conducted in Kausambi district, Allahabad, and compare the pattern with similar studies in literature.

## Materials and Methods

All cases attending a two day multispecialty medical camp in October 2005 in the rural interiors of Kausambi district, Allahabad, UP were included in the study. Patients with dermatological complaints were screened separately. A brief relevant clinical history was elicited, and a thorough dermatological examination was performed. Skin diseases were clinically diagnosed on the basis of classical clinical morphology in absence of investigative facilities in the rural medical camp. All skin diseases were classified into two groups, viz. infective and noninfective diseases. Those cases requiring specialized investigations for aiding diagnosis were referred to the Department of Dermatology, MLN Medical College, Allahabad, and were excluded from the study.

## Results

A total of 6639 persons were screened during the two day camp. Of them, 522 (7.86%) persons presented with skin diseases. Of these, 325 (62.2%) were males and 197 (37.8%) were females. The majority of the population was in the age group of 11–20 years with 164 cases (31.4%), followed by 21–30 year group with 101 cases (19.3%), 31–40 year group with 76 cases (14.6%), < 10 year group with 67 cases (12.8%), 41–50 year group with 59 cases (11.3%), 51–60 year group with 39 cases (7.5%) and > 60 year age group with 16 cases (3.1%) (Table 1).

**Table 1:** Age and sex distribution of cases

Age groups (years)	Male	Female	Total
< 10	38	29	67 (12.8)
11–20	112	52	164 (31.4)
21–30	62	39	101 (19.3)
31–40	45	31	76 (14.6)
41–50	33	26	59 (11.3)
51–60	24	15	39 (07.5)
>60	11	05	16 (03.1)
Total	325 (62.8)	197 (37.2)	522 (100)

Figures in parentheses are in percentages

A total of 580 dermatoses were found in the 522 persons screened. The number of dermatoses exceeded the number of persons as some had more than one skin disease. Infective conditions (343 cases, 59.1%) outstripped the prevalence of noninfective conditions (237 cases, 40.9%). Among the infective conditions, fungal infections were the most common disease with 187 cases (54.52%), followed by scabies in 79 cases (23.03%), bacterial infections in 58 cases (16.9%), leprosy in 13 cases (3.8%) and viral infections in 6 cases (1.75%). Among the fungal infections, *Tinea cruris* was found in 80 cases (42.8%), followed by *Pityriasis versicolor* in 36 (19.3%) and *Tinea corporis* in 33 (17.6%) cases (Table 2).

**Table 2:** Distribution of infective dermatoses

Diseases	Number (%)
Fungal	
Tinea cruris	80
Tinea corporis	33
Tinea capitis	12
Tinea unguium	09
Tinea pedis	06
Tinea manuum	05
Tinea faciei	01
Candidiasis	05
Pityriasis versicolor	36
*Total fungal*	187 (54.52)
*Bacterial*	
Folliculitis	36
Sycosis barbae	02
Other pyodermas	20
*Total Bacterial*	58 (16.9)
*Viral*	
Warts	05
Herpes simplex	01
*Total viral*	06 (1.75)
*Protozoal*	
Scabies	79 (23.03)
*Mycobacterial*	
Leprosy	13 (3.8)
TOTAL	343 (100)

Among the noninfective cases, eczemas were the commonest disorder in 93 cases (39.2%), followed by acne in 42 cases (17.7%), pigmentary disorders in 33 cases (13.9%) and polymorphic light eruptions (PMLE) in 32 cases (13.5%). In the eczema group, nummular eczema was found in 35 cases (37.6%), followed by hand eczema in 19 cases (20.4%), hyperkeratotic plantar eczema in 10 cases (10.8%), airborne contact dermatitis in 9 cases (9.7%), irritant/allergic contact dermatitis (ICD/ACD) in 7 cases (7.5%), atopic dermatitis in 5 cases (5.3%), seborrheic dermatitis and Pityriasis alba in 4 cases each (4.3%). Among the pigmentary disorders, vitiligo was observed in 21 cases (63.6%) and melasma in 12 cases (36.4%). Miscellaneous conditions comprised of 25 (15.7%) cases as follows: Urticaria – 9, pruritus – 5, milia and cutaneous maculopapular amyloidosis – 2 each, and *Ichthyosis vulgaris*, discoid lupus erythematosus, miliaria, oral aphthous ulcers, verrucous epidermal nevus, *Pityriasis rosea*, lichen sclerosus chronicus – 1 each (Table 3).

**Table 3:** Distribution of noninfective dermatoses

Diseases	Number (%)
Eczema	
Nummular eczema	35
Hand eczema	19
Atopic dermatitis	05
Seborrhoeic dermatitis	04
ICD/ACD	07
Airborne contact dermatitis	09
Hyperkeratotic plantar eczema	10
Pityriasis alba	04
*Total eczema*	93 (39.2)
Pigmentary	
Vitiligo	21
Melasma	12
*Total pigmentary*	33 (13.9)
Others	
Acne vulgaris	42 (17.7)
PMLE	32 (13.5)
Miscellaneous	25 (15.7)
TOTAL	237 (100)

## Discussion

The pattern of skin diseases in India is influenced by the developing economy, level of literacy, social backwardness, varied climate, industrialization, access to primary health care, and different religious, ritual and cultural factors.

The prevalence of skin diseases in the general population has varied from 6.3% to 11.16% in various studies.^2^–^7^,^11^ Our finding of 7.86% fell within this range. Although some studies have reported male preponderance,^3^,^5^ as in our study, others have reported female preponderance.^4^ Patients in their second and third decades formed the largest group of population in our study (50.7%), as in others, with prevalence rates ranging from 37% to 51.17% in similar age groups.^3^,^4^,^9^

The prevalence of infective disorders has outstripped that of noninfective disorders in some studies, varying from 42.68% to 89.72%.^3^,^4^,^9^,^10^ Further, this trend was noticed in our study. However, some other studies have reported a higher prevalence of noninfective disorders, varying from 53.15% to 58.07%.^2^,^5^–^8^ This variance could possibly be due to differing susceptibilities in different population groups in diverse geographical regions.

Among the infective conditions, while fungal infections was the most common disorder in most studies, varying in prevalence from 12.8% to 46.25%,^3^–^5^,^7^–^11^ pyodermas were reported as the largest group in isolated studies.^6^,^12^ Higher prevalence of fungal infections is attributed to hot and humid climatic conditions in some geographical regions. The prevalence of scabies has varied from 8.56% to 16.0% in some studies.^5^–^9^ Our study reported a relatively higher, but statistically insignificant, prevalence of fungal infections (*P* < 0.2) and scabies (*P* < 0.2) compared with other population groups. The prevalence of leprosy in our study (3.8%) conformed to the reported range of 1.7% to 11.7%.^2^,^3^,^5^,^6^

Among the noninfective conditions, eczema has been reported as the largest group, the prevalence varying from 16.17% to 33.93%.^3^–^11^ Our study reported a relatively higher, but statistically insignificant, prevalence of eczemas (*P* < 0.2) as compared to other studies. Contact dermatitis was the commonest form of eczema (22.69%–23.54%) in various studies, vis-à-vis ours, owing to occupational variation.^6^,^9^,^11^ On the other hand, nummular eczema, the commonest eczema in our study, was prevalent in merely 3.3%–3.9% of these studies. Peculiarities of geographical and occupational variation have always effected the expression of skin diseases. Cold injuries, reported in 7.1% of population in Kashmir region, have never been reported in other studies including ours.^10^

To conclude, our study confirmed a higher prevalence of infective dermatoses than noninfective dermatoses. A relatively higher, but statistically insignificant, prevalence of fungal infections, scabies and eczemas were observed, which probably reflects the minor regional variance in our study group. Our study was conducted on a large sample of population in a community setting. Hence, it may not suffer from referral bias and therefore promises to be a true representation of the point prevalence of dermatological disorders in the local population at a regional location.
